# Feasibility of Implementing a Patient-Centered Postoperative Wound Monitoring Program Using Smartphone Images: A Pilot Protocol

**DOI:** 10.2196/resprot.6819

**Published:** 2017-02-22

**Authors:** Sara Fernandes-Taylor, Rebecca L Gunter, Kyla M Bennett, Lola Awoyinka, Shahrose Rahman, Caprice C Greenberg, K Craig Kent

**Affiliations:** ^1^ Wisconsin Surgical Outcomes Research Program University of Wisconsin Madison, WI United States

**Keywords:** telemedicine, smartphone, surgical site infection, transitional care, feasibility study, clinical research protocol

## Abstract

**Background:**

Surgical site infections (SSI) represent a significant public health problem as the most common nosocomial infection and a leading cause of unplanned hospital readmissions among surgical patients. Many develop following hospital discharge and often go unrecognized by patients. Telemedicine offers the opportunity to leverage the mobile technology to remotely monitor wound recovery in the transitional period between hospital discharge and routine clinic follow-up. However, many existing telemedicine platforms are episodic, replacing routine follow-up, rather than equipped for continued monitoring; they include only low-risk patient populations and those who already have access to and comfort with the necessary technology; and transmit no visual information.

**Objective:**

Drawing upon the Coleman model for care transitions and the Proctor model for implementation, we propose a protocol of postoperative wound monitoring using smartphone digital images. In this study, we will establish the feasibility of such a program, both for patients and for the clinical care team.

**Methods:**

We will recruit 40 patients or patient/caregiver pairs from our inpatient vascular surgery service. Eligible patients will be English-speaking, 18 years of age or older, and have an incision at least 3 cm in length. Participants will receive a training session, during which they will learn to use the device and the wound monitoring smartphone app. Following hospital discharge, they will submit digital images of their wound and responses to a survey about their recovery for 14 days. Experienced health care providers on the vascular surgery inpatient service will review transmitted data daily and contact patients for any concerning findings.

**Results:**

Primary outcomes will include participant adherence to the protocol, time required for providers to review submissions, time from submission to provider review, and participant satisfaction. Secondary outcomes will include SSI detection and hospital readmission.

**Conclusions:**

Health systems are increasingly dedicating efforts to transitional care improvement programs. This feasibility trial will confirm whether patients and their caregivers can learn to use a postdischarge wound monitoring smartphone app and will assess patient and provider satisfaction. This protocol will provide preliminary evidence for a shift in the delivery of postdischarge care in a patient-centered and cost-effective manner.

**Trial Registration:**

Clinicaltrials.gov NCT02735525; https://clinicaltrials.gov/ct2/show/NCT02735525 (Archived by WebCite at http://www.webcitation.org/6oIvN4Mab)

## Introduction

### Background

Surgical site infection (SSI) represents a significant public health problem as the most common nosocomial infection and the leading cause of unplanned hospital readmissions among surgical patients [1-4]. SSI can be intractable because up to 84% develop in the critical interval between hospital discharge and routine follow-up [1,5]. Moreover, since the majority of patients have little experience caring for a surgical wound, they rarely recognize early stage wound infections and often present with an advanced infection that requires rehospitalization [6,7]. Conversely, SSI diagnosed at an early stage can be treated in the outpatient setting with oral antibiotics and wound care, precluding the need for readmission, intravenous antibiotics, and reintervention. The fact that SSI develops or progresses in the outpatient setting makes transitional care coordination a promising area of focus in the management of SSI.

Hospitals are incentivized to improve transitional care for surgical patients as the Center for Medicare and Medicaid Services (CMS) increasingly imposes financial penalties for unplanned readmissions after surgery through the Readmissions Reduction Program as part of the Patient Protection and Affordable Care Act [8]. However, transitional care coordination following surgical procedures has received less attention from researchers and hospital systems relative to medically managed conditions [9,10]. Although the Centers for Disease Control and Prevention have long monitored SSI rates through the National Healthcare Safety Network, the majority of prevention efforts occur during the operative procedure and any associated inpatient stay; few early detection and prevention efforts crosscut care settings. We leverage the alignment of national policy and gaps in existing SSI surveillance to implement a patient-centered mobile health (mHealth) intervention focused on stemming the burden of SSI and readmissions through image capture of wound healing.

Telemedicine, and mHealth specifically, offers an opportunity to leverage technology for the remote monitoring and early detection of SSI during the transitional care period, including wound monitoring using smartphone digital images [11]. However, significant barriers have restricted adoption of telemedicine, particularly in the United States where regulatory and reimbursement policies present unique challenges. The few studies examining continued monitoring of recovery following hospital discharge have been performed outside of the United States [12-14] and have not adhered to privacy standards set forth by the Health Insurance Portability and Accountability Act (HIPAA) [15]. Moreover, sustainable implementation of mHealth protocols for wound monitoring requires that they support existing patient-provider-caregiver relationships, fit into patients’ daily routines following hospital discharge, and provide visibility of the wounds’ healing for both patients and providers [16]. Therefore, the design of a wound monitoring protocol using telemedicine must support both patients and providers in their existing roles without creating a significant burden.

Another essential feature of such a protocol is scalability to a full patient panel, particularly with respect to novice-user patient populations. The cost effectiveness and widespread accessibility of a telemedicine protocol for wound monitoring can help to ensure scalability. Specifically, the cost per enrolled patient must be sufficiently low to justify targeted enrollment among patient populations who are at high risk of developing a complication requiring readmission following discharge in the context of quality-based payment and financial penalties for readmission. In addition, health systems must allocate resources to ensure that patients who have no experience using smartphones are empowered and equipped to participate. A majority of published studies rely on self-selection into telemedicine, conferring “digital access” to technologically savvy patients who tend to be younger, more educated, and wealthier [11]. Telemedicine thus has demonstrated potential to exacerbate existing access and utilization care disparities, and dedicated resources are required to mitigate this gap.

### Objectives

#### Overview

We propose to address identified shortcomings in published mHealth studies with a patient-centered, mHealth outpatient wound surveillance program designed to promote early recognition of SSI following discharge. The current trial is part of a larger project funded by Agency for Healthcare Research and Quality R21 HS023395. The goals of this larger project are (1) to empower patients, particularly novice smartphone users, to be mindful of their wounds and to partner with their surgeons in monitoring their postoperative recovery; (2) to diagnose SSI, when it occurs, at an early stage, enabling outpatient management; and (3) to prevent hospital readmission and the serious morbidity and mortality associated with wound complications. The project entails teaching vascular surgery patients to use a simple, linear smartphone app to take and transmit images of their postoperative wounds and symptom information (as yes/no questions) daily for the first 2 weeks following hospital discharge; surgical staff then review the submissions to discern the presence of a complication.

Prior work from our group and others has demonstrated enthusiasm from patients and their families regarding participation in transitional care programs [17]. In addition, we have shown that digital images are sufficient for diagnostic and therapeutic decision making, resulting in decisions comparable to those based on in-person evaluation [18]. Using an internally developed smartphone app with an accompanying training program grounded in tenets of adult learning and memory retention, we have also demonstrated that patients and their caregivers can learn to use the app with a high level of independence and satisfaction [19].

The next step in the project is to pilot test the full patient-centered outpatient wound surveillance program to establish its feasibility for both patients and the service line of a large academic tertiary care institution. With a targeted enrollment of 40 patients, outcomes will include evaluation of the module’s technological capability, including (1) barriers to participation, (2) patient attrition/adherence, (3) picture and information quality, (4) successful information transmission and assimilation into clinical workflows, (5) ease of integration into the clinical service line, and (6) the ability of health care professionals to identify early wound infection from photographs.

#### Theoretical Foundation

**Figure 1:**
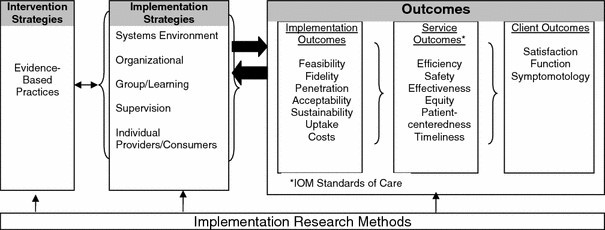
Conceptual model of implementation research. Reprinted with permission from Procter et al [20].

We use Proctor’s model of implementation research to structure the evaluation of our feasibility trial (Figure 1) [20]. This framework integrates 3 bodies of theory, including multilevel organizational models of change, stage pipeline models of implementation that accelerate interventions from development to real world practice settings, and structure-process-outcome models of health services use. This model is particularly suited to our project because our intervention cross cuts care settings (inpatient, outpatient, and home), requires integration at multiple organizational levels (clinical, patient/caregiver, information technology infrastructure, and leadership), and necessitates real-world feasibility testing at bedside prior to widespread implementation. Accordingly, this framework facilitates defining outcomes on different organizational levels: implementation, service, and client. Although this model is largely a heuristic model, it is useful here because our trial is a small, research-focused feasibility trial rather than a large-scale implementation study involving widespread uptake of an established intervention. Moreover, Proctor’s emphasis on diffusion of innovation theories lends itself to studying a new mHealth technology in a clinical setting.

Interdisciplinary collaboration and stakeholder engagement are fundamental to this model. During intervention and app development, we engaged patient advocates, physicians, surgical nursing staff, and community members to provide structured feedback via focus groups and interviews on app content and ancillary training materials to ensure that all functionalities and language used are clear and consistent with discharge instructions. Additionally, formal usability tests involving 9 postoperative vascular surgery patients at our institution established acceptance by the target population and patients’ and their caregivers’ capacity for completion after a short training session, with a median training time of 8.5 minutes and an average System Usability Scale score of 83.3 [19]. We also involved surgical leadership, surgeons, nurse practitioners, physician assistants, information technology personnel, and nurses in the vascular surgery inpatient unit to develop clinical implementation strategies that can be easily integrated into their daily workflow. Defined outcomes, described in detail in the Methods section, are mapped onto 3 organizational levels: implementation, service, and client outcomes.

#### Objectives and Hypotheses

Our overall objective is to evaluate the feasibility and effectiveness of a protocol for postdischarge wound monitoring using a smartphone app and its ability to detect postoperative wound complications and reduce hospital readmissions in a vascular surgery patient population. In this phase of the larger project, we focus on feasibility of the protocol from both the patient and the provider perspectives. We hypothesize the following:

1. Patients can be engaged in their postoperative care by participating in a smartphone protocol to monitor wound healing, completing the protocol daily for 2 weeks.
2. Reviewing received submissions and acting on any detected abnormalities can be integrated into existing service lines without overburdening clinical staff.

## Methods

### Participants

We will recruit 40 patients or patient/caregiver pairs for participation in our pilot study, aiming for 20 independent participants and 20 patient/caregiver pairs, in an effort to capture all eligible patients over the study duration based on our institution’s annual volume. Recruitment will occur primarily in the University of Wisconsin Hospital and Clinics (UWHC) vascular clinic at the time of consent for surgery and the postoperative inpatient vascular surgery service; UWHC performs more than 600 inpatient vascular procedures annually, a majority of which are open rather than endovascular [21]. As a major referral center for the state of Wisconsin, our institution performs a relatively high number of nonelective procedures necessitating additional recruitment in the postoperative period before hospital discharge. English-speaking patients over the age of 18 with an incision at least 3 cm in length will be eligible. All eligible patients will be approached for participation in a consecutive fashion. Patients and their caregivers will receive training to learn to use the smartphone itself as well as the wound monitoring app during their stay with a refresher session on the day of discharge including a test image transmission prior to discharge. This will allow for reinforcement of the initial training session as well as the acquisition of a baseline image for later comparison. Smartphones will be provided for use during the study, which participants will be able to keep as remuneration after study completion.

Participants will be enrolled in waves of approximately 5, with time between waves for protocol evaluation and to identify areas for intervention improvement. Patients who are approached but decline to participate will be asked to provide their reason for declining.

### Ethics Approval and Consent to Participate

The University of Wisconsin Institutional Review Board (IRB) approved this study (UW IRB #2015-1581). All future modifications to the protocol or to the consent process will go through this IRB for review and approval.

Participants (and their caregivers, where applicable) will be approached for informed consent in person during the postoperative, predischarge period. Eligibility will be determined by medical record review and consultation with service providers. Proxy consent will be obtained for participants who are unable to participate if a competent family member or caregiver can be identified. Participants will receive copies of all signed consent forms.

### Intervention

**Figure 2:**
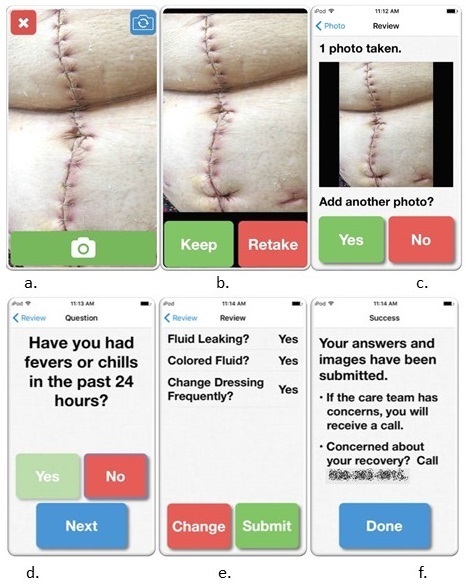
WoundCheck iOS app: image taking and survey sample screenshots. Picture from Shutterstock.

WoundCheck is an internally developed and user-tested iOS app that enables patients to transmit daily wound images and symptom information from their home or postacute care facility to the surgical care team in the hospital (Figure 2) [19]. There are 2 phases of the app: an image-taking phase where patients take up to 4 digital images of their surgical wound and a brief survey of 6 yes/no questions regarding recovery (Textbox 1). Survey questions were developed based on prior work from our group validating smartphone digital images for postoperative wound monitoring and were designed to capture information not as easily appreciated from the submitted images [18]. Information generated from the app is automatically transmitted to our research server as the final step of the app. The app, which features large font, large buttons, and simple language and design, is accompanied by a training program that is delivered in person prior to discharge along with a written instructional packet.

**WoundCheck survey module yes/no questions.**

- Have you had fevers or chills in the past 24 hours?
- Have you changed how you take your medication in the last 24 hours?
  - If yes, is this change related to your pain medication?
  - If yes, did you increase your pain medication?
- Has the area around your wound become red in the past 24 hours?
- Has the area around your wound become swollen in the past 24 hours?
- Is there a bad smell coming from your wound?
- Is fluid leaking from your wound?
  - If yes, is the fluid white, yellow, or green?
  - If yes, do you change your dressing more than once per day because fluid soaks through?

We developed the app using the Coleman model transitional care framework to guide content [22-24]. The Coleman model is specifically designed to reduce discontinuity in care transitions and addresses the needs of elderly patients and patients with complex, chronic conditions [25]. Moreover, it focuses on patients and caregivers as the “common thread linking differing providers and settings,” emphasizing patient education and empowerment as essential for facilitating (1) medication self-management, (2) use of a dynamic patient-centered record, (3) completion of follow-up care, and (4) knowledge of red flags that could indicate a worsening of their condition. Our smartphone-based intervention is designed to promote mindfulness of the wound, identify red flag symptoms and medication misuse, and direct and document communication between the patient/caregiver and the surgical service.

At the time of recruitment, we will introduce patients and any caregivers to the smartphone and teach them to complete the app independently, a process that takes between 3.9 and 23.0 minutes, based on our preliminary results from usability testing [19]. We developed the training drawing on the following tenets from the adult learning and memory literature: the need for repetition and multiple formats of educational materials [26], the decline in motivation when not experiencing success [27] or when the purpose of the task is not clear or relevant [28], the need for active engagement with teach-back [29], and the importance of letting the learner set the pace of learning [30].

**Figure 3:**
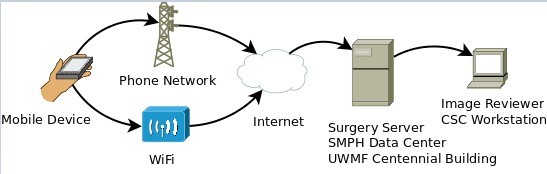
WoundCheck workflow.

The patient will take a baseline digital image on the day of discharge to serve as a reference for future comparison. Participants will be asked to transmit a digital image and complete the survey regarding their recovery within the app once per day for 14 days. For those patients discharged to a postacute care facility, the participant or their specified caregiver will still be responsible for submission. We will not rely on the staff of the facility to complete the protocol. Transmissions will be sent through an encrypted connection to a secure research server, a process which has been designed to be HIPAA compliant (Figure 3). Once transmitted, the images are no longer stored on the device.

Participants are provided an iPhone 5S that is theirs to keep at the end of the study. At the time of training, they are counseled that the cost of the phone and the data plan are paid for through the research study, but they are asked not to use the phone for other purposes for the duration of the study protocol. They are also counseled that this protocol of wound monitoring is a supplement to usual care rather than a replacement. As such, they are given the number to our vascular surgery clinic and encouraged to communicate with them as they would outside the study protocol. During training, participants are told that the nurse practitioners (NPs) will review the submissions daily, usually at the end of their workday in the later afternoon. Phone calls from the NPs to participants for concerning findings will likely be made at that time. If a participant has a concern and either does not receive a phone call or does not wish to wait to receive one, they are encouraged to call the clinic or the research contact. Contact information for the vascular surgery clinic and study personnel is provided at 2 points in the app itself and in the provided instructional packet so that patients and their caregivers can easily call with questions or concerns.

Each afternoon, at a time designated by clinical personnel as most accommodating of existing clinical workflows, a vascular surgery service NP or researcher with MD surgical training will review the transmitted images as well as the responses to the survey questions within the app. A short form checklist documenting the appearance of the surgical wound will be completed for each image received [18]. This checklist was previously developed and validated by our research group, drawing upon definitions of surgical site infection and other wound complications from the Centers for Disease Control and Prevention and measures compiled by Cutting and colleagues [31,32]. The checklist also includes a question regarding whether the image is adequate for evaluation, and if an image is found inadequate, space is available to explain why (eg, insufficient light, out of focus, parts of the wound excluded from view). The time required to review the images will be recorded as will the time between submission and review. Any concerning findings prompt a phone call from the surgical service NP to the patient to gather more information and recommend additional intervention/treatment as indicated, which may include antibiotics or a clinic visit. If there is uncertainty about evaluating or interpreting submitted information, the NP will contact the operating surgeon or the vascular surgeon on call to discuss concerning findings.

If a participant has not submitted information for 24 hours, research staff will phone him or her to troubleshoot barriers to completion; these unscheduled calls will be recorded for analysis. Similarly, if the digital images are inadequate for diagnostic purposes, research staff will contact the participant to remind them the goals for the image and help them strategize how to take an effective image on their subsequent submissions. Calls will not be punitive but aimed at minimizing study attrition, identifying reasons why patients are unable to complete the protocol, and identifying possible measures to improve the protocol. Additionally, all participants will receive a phone call on postdischarge day 6 to assess use of the app and continued willingness to participate. A final phone call will be made at the end of 2 weeks, when the participant has completed submissions, to evaluate satisfaction with the protocol. The details of these phone calls will be recorded on a secure data collection spreadsheet. The feasibility trial for the intervention is registered at ClinicalTrials.gov [NCT02735525].

### Implementation

#### Organization

Multilevel organizational implementation strategies include information systems implementation, legal compliance, clinical integration, and administrative engagement. To that end, we engaged stakeholders within hospital and surgical leadership to ensure that our mHealth intervention is consistent with existing quality improvement initiatives within the hospital and can be used to augment existing transitional care programs; during this process, we were able to garner enthusiasm for the intervention among clinical and administrative leadership. We also engaged hospital information systems personnel to verify that the hospital had not already licensed a software platform that we could modify for our intervention and to explore the possibility of future medical record integration. Our human subjects review process mandated legal review of app security, cloud-based workflow, and electronic data storage in conjunction with our cybersecurity department to ensure HIPAA compliance. Finally, we solicited input from surgeon-scientists in the Wisconsin Surgical Outcomes Research Program and the Institute for Clinical and Translational Research to leverage existing infrastructure to support clinical evaluation and clinical trials in the department of surgery.

#### Group/Team

We opted to assess intervention feasibility on the vascular surgery service line within the department of surgery to assess clinical implementation and workflow for a defined patient population with (1) a high base rate of postoperative SSI and readmission, (2) a high number of novice smartphone users, and (3) a high number of regional referrals (making travel for postoperative care potentially burdensome to the patient). Vascular surgery is comprised of a patient population with the highest readmission rate among surgical specialties, most commonly for SSI [3,21,33]. Vascular surgery also has the highest projected demand growth among medical specialties (31% by 2025) after adjusting for expanded coverage under the Patient Protection and Affordable Care Act due to the aging US population and the prevalence of underlying disease burden, such as diabetes [34].

Our vascular surgery service line is comprised of 10 board certified vascular surgeons, 3 inpatient NPs, and a team of nurses and outpatient physician assistants. We engaged the vascular surgery division chief and the service NPs early in the intervention design process to make sure the protocol addressed priority areas of transitional care and to ensure clinical buy-in. Interviews with these clinical stakeholders and ongoing feedback ensured that the intervention is consistent with service line quality improvement efforts and is integrated into existing workflows, particularly that of the NPs undertaking daily wound image review, as smoothly as possible. We presented the proposed protocol at clinical and quality improvement standing meetings and made additional adjustments to the protocol based on feedback. We also notified our 12 regional clinics about the protocol to make sure participants with questions who called the clinic could be directed to the research and app support contact. Whereas participant recruitment, image review, and any follow-up resulting from anomalous findings in the images will take place in the clinical setting, protocol support, materials sourcing and design, teaching patients to use the app, and phone calls associated with the protocol will be performed by 3 research staff.

#### Individual Providers/Consumers

Given the high prevalence of novice users in the vascular surgery patient population, vetting app and training content in focus groups as well as subsequent formal usability testing provided essential foundations for implementation. The use of published user interface standards (International Organization for Standardization 9241-12) similarly facilitated ease of use [35]. Leveraging state-negotiated rates for mobile phones, we purchased generation-old iPhone 5Ss at a cost of $0.99 per unit and provided the phone as remuneration for study participation to remove the burden associated with having to return the phones. Study service plans cost $34.50 per month per patient, bringing estimated materials costs to $53.50, including a protective phone case and written training materials. Essential features of patient-level implementation include conducting training during the postoperative inpatient stay; this allows us the attention of the patients and assessment of their capacity, caregiver interaction, and engagement during both the inpatient and outpatient settings.

### Outcomes

We have defined our outcomes in 3 domains: implementation outcomes, service outcomes, and client outcomes. The primary, multidimensional outcomes of interest are protocol completion by participants and the burden of the protocol on clinician workflow. Our implementation outcomes include patient adherence to the protocol, cost, clinician/service investment, and technological system compatibility. Service outcomes include the incidence of advanced stage SSI diagnosis, readmissions, and service integration (patient/caregiver participation and follow-up by clinical staff). Client outcomes include satisfaction measured using both previously validated questionnaires and internally developed project-specific measures. We describe our specific measures below.

Implementation outcomes include patient adherence, cost, clinician/service investment, and technological system compatibility. We will calculate the number of eligible patients approached to participate in the protocol relative to the number who consent to participate. Reasons for declining to participate will be recorded. We will also report the percentage of patients who completed the app for the full length of the protocol (daily from the day of discharge until the day of scheduled clinic follow-up) without requiring a reminder phone call. Likewise, the percentage of participants who required a reminder phone call when they did not submit an image or their survey responses within a 24-hour period will be recorded, as well as their reasons for not completing the protocol. The number of phone calls required to reach the participant will be logged, as will the day the phone calls were made. Participant sociodemographic information and relevant comorbidities will be collected from the medical record to ensure that the protocol was accessible to a diverse patient population. We will also identify participant characteristics that would preclude full participation in the absence of a caregiver.

We will calculate the per-patient cost of the protocol including device and plan cost, ancillary materials costs (phone use, written training materials, iPhone cases), and researcher/clinical person-hours spent on the protocol. To determine the burden of the protocol on the clinical workflow, we will determine the time required to complete the review of submitted images and survey responses by the service NPs or study personnel. The time required to make follow-up phone calls will also be recorded. Finally, we will evaluate the data assimilation and review process. To ensure that the service NPs or research personnel review transmitted information within a clinically appropriate timeframe, we will measure time from receipt of transmission to diagnostic review and the time from diagnostic review to follow-up call to the patient, when indicated. Upon trial completion, we will ask all participating clinical staff to anonymously complete the Patient-Centered Care Improvement Guide’s Self-Assessment Tool and to provide open-ended feedback on the protocol, their perceptions of its utility, and areas for improvement for long-term sustainability [36]. Finally, we will ask NPs whether they would prefer medical record integration of the app images and content or whether they find our custom provider review interface better suited to their practice.

Service outcomes include late stage SSI diagnosis, readmissions (using a modified CMS definition of any unplanned recurrent admission to an acute care facility before routine follow-up in the 30 days following discharge, subject to certain exclusions including same-day readmission and discharge against medical advice), and service integration (patient/caregiver participation and follow-up by clinical staff) [37]. Although the design and sample size are insufficient to evaluate a significant change, we will track the percent of SSI diagnosed at an early stage (ie, managed on an outpatient basis) and the percent of readmissions; specifically, any late stage SSI diagnosis or unplanned readmission among participants will be thoroughly evaluated for process/diagnosis failures. We will also record the percentage of patients who require intravenous antibiotics or surgical reintervention and whether these patients missed 1 or more of their daily submissions.

To assess client outcomes associated with patient and caregiver perceptions, we will ask participants to complete 2 established, validated scales: the Care Transitions Measure from the Coleman Care Transitions Program and the “After surgery” questions from the Consumer Assessment of Healthcare Providers and Systems (CAHPS) Surgical Care Survey during the phone call made at the end of the protocol [22,38]. The Coleman Care Transitions Program was conceived through the use of focus groups and validated in a large randomized controlled trial among older adults; the CAHPS Surgical Care Survey is a National Quality Forum–endorsed measure of the patient experience following surgery. During the phone call at the end of the protocol, open-ended qualitative interviews will be performed to evaluate participant satisfaction and to elicit feedback regarding barriers to protocol completion and suggestions for possible areas of improvement. Our preliminary goal for patient adherence for the study period is 90% over the first week and 70% over the full 2 weeks; all participants will be queried for barriers to adherence during a routine day 6 follow-up phone call. Our goal for diagnostic quality of all photos submitted, as judged by reviewing clinicians, is 95%. Any instances of photos not being reviewed within 24 hours of transmission will be investigated for process failures.

We will also evaluate the success of the training protocol and participant satisfaction and confidence following training. We will record the time required to successfully complete the training module and patient/caregiver ability to independently complete the app following the training session. We will collect sociodemographic information and smartphone experience from participants, in part to evaluate subgroups of patients whose training needs are unmet by the protocol. Any redesign of the training module will be based on the questions asked and evaluation of the module. At the end of training, we will elicit free response written feedback on the training module from all participants. Responses will be cataloged and content analysis will evaluate themes, pitfalls, and potential barriers to implementation. The training module will be redesigned iteratively based on these results.

### Data Confidentiality and Access

All medical record information and study devices will be stored in a secure, locked research office or on a secure server in the department of surgery. When possible, identifiable information will be kept separately from information that is not readily identifiable, and the 2 will be linked with a randomly generated identifying number. Digital images and symptom information collected through the smartphone app will be stored in the department of surgery server behind a firewall; none of this information will be stored on the smartphone on which it was generated.

All smartphones will be password protected, allowing only patients and their caregivers access to the phone. Transmitted data will include only the study identifier, digital images of the wound, and symptom information; there will be no personal health information recorded in the transmission itself. Upon protocol completion, the service plan will be discontinued, and participants will be given instructions to return the phone to its original factory settings. Participants can keep the erased phone for personal use.

All of our data security measures will be fully outlined in all recruitment and consent materials.

### Dissemination

Results of this feasibility trial will be disseminated through peer-reviewed publications as well as at scientific conferences. Lay summaries and presentations at standing meetings will provide feedback to clinical and administrative stakeholders in the hospital. Our results will inform a larger multi-institutional trial using this app as part of a larger transitional care intervention aimed at readmission reduction among complex vascular and colorectal surgery patients.

## Results

This feasibility trial began enrolling patients in June 2016. Enrollment and data collection continued until 40 patients completed the protocol, with study completion in December 2016.

## Discussion

### Summary

As the medical community has increasingly dedicated its efforts to improving transitions of care, particularly transitions from an inpatient stay back to the community, the majority of solutions involve assigning patients a discharge advocate, transition coach, or nurse case manager to provide close follow-up during the transition [22,39]. However, while these methods are effective in reducing hospital readmissions in medical patients, they have frequently not included the crucial visual component needed to fully monitor surgical patients in the postoperative period. The rise of smartphones capable of transmitting visual information and their increasing market penetration have generated enormous opportunities for the incorporation of mobile devices as extensions of care traditionally provided in person.

Through prior work, we have demonstrated that patients and their caregivers are accepting of a protocol of postdischarge monitoring using smartphone technology, that digital images can be used to make diagnostic and therapeutic decisions comparable to those made in person, and that patients and their caregivers can learn to use the postdischarge monitoring app after a short training session tailored to their needs. This feasibility trial will confirm whether patients can complete a smartphone protocol and assess patient and provider satisfaction. In an era of shortened hospital lengths of stay and increasing penalties for higher than expected readmission rates, this protocol provides preliminary evidence for changing the way postdischarge care is delivered with the goal of providing it in a cost-effective manner.

### Limitations

This is a feasibility study without a control arm and thus cannot draw conclusions in reference to usual care. Although we will be enrolling only vascular surgery patients, we feel confident that if a patient population that is largely elderly with limited technology exposure can complete the protocol, it can be generalized to a wider surgical population with some caveats. Specifically, our surgical population is racially and culturally homogeneous (over 95% white) and largely comprised of native English speakers. In addition, we are pursuing implementation on a single specialty service and cannot discern whether our intervention scales to a larger service or surgical care teams with different organization of services. Moreover, our hospital has substantial information technology and nursing resources to support our intervention, and significant changes to the protocol might be required for implementation at a hospital with limited resources.

## Abbreviations

CAHPS: Consumer Assessment of Healthcare Providers and Systems
CMS: Centers for Medicare and Medicaid Services
HIPAA: Health Insurance Portability and Accountability Act
mHealth: mobile health
NP: nurse practitioner
SSI: surgical site infection
UWHC: University of Wisconsin Hospital and Clinics
